# Deciphering the Neighborhood Atlas Area Deprivation Index: the consequences of not standardizing

**DOI:** 10.1093/haschl/qxad063

**Published:** 2023-11-03

**Authors:** Stephen Petterson

**Affiliations:** Robert Graham Center, Washington, DC 20036, United States

**Keywords:** health inequity, area measures of deprivation, payment reform

## Abstract

The Area Deprivation Index (ADI) is a widely used measure recently selected for several federal payment models that adjusts payments based on where beneficiaries live. A recent debate in *Health Affairs* focuses on seemingly implausible ADI rankings in major cities and across New York. At the root of the issue is the importance of standardization of measures prior to calculating index scores. Neighborhood Atlas researchers are implicitly arguing that their choice to not standardize is of little consequence. Using the same data and methods as the Neighborhood Atlas, this paper focuses on this choice by calculating and comparing standardized and unstandardized ADI scores. The calculated unstandardized ADI nearly perfectly matches the Neighborhood Atlas ADI (*r* > 0.9999), whereas the correlation with a standardized version is much lower (*r* = 0.7245). The main finding is that, without standardization, the ADI is reducible to a weighted average of just 2 measures—income and home values—certainly not the advertised multidimensional measure. Federal programs that have incorporated the ADI risk poorly allocating scarce resources meant to reduce health inequities.

## Introduction

Health inequities across the United States are closely linked to social and economic disparities. Race, poverty, education, family structure, and joblessness, among other factors, are strong predictors of poor health and poor access to health care.^1,2^ The federal government has a long history of trying to identify communities with the greatest need to target resources and create incentives for health care providers to practice in those communities.^3-5^ Recent payment models developed by Centers for Medicare and Medicaid Services (CMS) build on these efforts. These new models, however, are facing growing criticism for including the Neighborhood Atlas Area Deprivation Index (ADI) as their indicator of community disadvantage. As an example, CMS's Medicare Shared Savings Program (MSSP) was changed for fiscal year 2023 to increase advance investment payments to Accountable Care Organizations (ACOs) when more beneficiaries assigned to the ACO are (1) dually eligible for Medicare and Medicaid, (2) enrolled in the part D low-income subsidy, and/or (3) live in an area with high deprivation.^6^ The last criterion is defined as being in the 85th percentile or higher using the ADI.^7^

A recent *Health Affairs* article found a very weak relationships between life expectancy at birth and ADI scores in several large cities—New York, Washington, DC, and San Francisco.^8^ With a few exceptions, they found that no block groups in New York City and none in Washington, DC, would qualify using the MSSP's 85th-percentile cutoff. A subsequent *Health Affairs* paper showed that the ADI yielded counterintuitive results in a regional examination of New York State.^9^ In New York City, block groups with high levels of deprivation, as measured, for example, by poverty, education, and family structure, had ADI scores placing them in the least-deprived deciles. With some exploration and confirmation from Neighborhood Atlas researchers, the authors of this paper concluded that, by failing to standardize variables before computing ADI scores, the Neighborhood Atlas inadvertently gives greater weight to indicators measured in dollars, particularly median home values. Assessing this evidence, Rehkopf and Phillips^10^ recommend that all studies that used the Neighborhood Atlas ADI “should be reinterpreted as capturing associations with median home values. It also means that current policy applications developed using the Neighborhood Atlas ADI need to reassess correlations that were tested and used to set payment weighting thresholds.”

Such a mistake with a prominent, widely used area measure is troublesome. What is surprising is that, in response to their critics,^11^ Neighborhood Atlas researchers doubled down and insisted that their version of the ADI remains sound:

Strong statements made in opinion pieces are just that—opinions without a well-validated and thoroughly evaluated alternative solution. They do not outweigh nearly 1000 peer reviewed scientific articles. It is therefore critically important to rely on the science and validation of the tool itself, which is the best proxy for scientific rigor.

In this response, there is no attempt to demonstrate that their critics are wrong, nor compare an ADI that standardizes values to one that does not. Instead, they appear to assume that not standardizing is of little consequence.

This paper focuses on 1 small piece of what it takes to develop a deprivation index by examining the issue of standardization. Rather than relying on the percentile scores available in the publicly available ADI data, I obtained the underlying American Community Survey (ACS) data for the 17 ADI measures to calculate raw composite scores using both standardized and nonstandardized values. With an illustration of how raw scores are calculated, the implicit weighting of each ADI measure using the Neighborhood Atlas approach becomes evident and quantifiable. Converting these raw scores into percentiles makes it possible to compare them with the Neighborhood Atlas’ national rankings.

## Background

In a 2003 paper, using data from the 1990 Decennial Census, Singh^12^ described a principal component analysis (PCA) that identified 17 measures that “indicated a theoretically and empirically meaningful clustering of the given indicators.” The ADI was created at 3 levels: census tract, ZCTA (Zip Code Tabulation Area), and county. The Neighborhood Atlas ADI^13^ is maintained and updated by the University of Wisconsin School of Medicine and Public Health but is only available for census block groups and 9-digit zip codes. While ZCTAs and counties vary substantially in population size, tract populations typically are between 1200 and 8000; tracts are made up of block groups that contain between 600 and 3000 people.^14^ Only percentiles at a national level and deciles at the state level are included in the publicly available data.

The Neighborhood Atlas documentation indicates that they used Singh's tract-level scoring coefficients combined with block-group–level data: “ADI scores [were calculated] for each US Census block group using Singh's methodology. This involved summing Singh's 17 Census indicators weighted by Singh's factor score coefficients for each indicator.”^15^ The available documentation does not include an examination of whether scoring coefficients obtained from a tract-level analysis using 1990 data are appropriate for constructing an area measure with 2020 block-group data. For my purposes here, I will try to adhere to the Neighborhood Atlas methodology for the sake of simplicity.

In a standard PCA, it does not matter if measures are standardized prior to running the analysis since most software packages convert a covariance matrix into a correlation matrix of standardized values prior to the factor analysis. Standardization does matter in calculating factor scores. Mathematically, scoring coefficients are obtained by regressing standardized values of the measures on predicted factor scores.^16-19^ The magnitude of the scoring coefficients reported by Singh clearly indicates that he used standardized values prior to obtaining factor scores (see Table A5).

Standardization is customary in multidimensional area measures of deprivation, socioeconomic status, and health. For instance, in the Robert Wood Johnson Foundation County Health Ranking technical documentation,^20^ the need to standardize is presented as obvious:

Our measures use different types of data as input, and when calculated, the measures use different types of metrics as output. Some measures use percentages, while others use rates, averages, or other metrics. Standardizing each of these measures transforms them to the same metric—a mean (average) value of 0 (zero) and a standard deviation (measure of spread) of 1.

According to Gilthorpe,^21^ “Standardisation is generally acknowledged as an equitable process for combining several variables. Without it, disproportionate scales and ranges will give undue prominence to some variables at the expense of others.”

## Data and methods

All 17 ADI variables are available at the block-group level from the ACS. I used 2020 data, pooled from 2016 to 2020, to match the 2020 Neighborhood Atlas ADI (see Supplement for discussion of the ACS measures, the corresponding Stata code, and the decision to use median household income instead of family income). To be consistent with the Neighborhood Atlas approach, I used scoring coefficients from Singh's 2003 tract-level analysis to calculate raw ADI scores. To address the problem of missing values, I retrieved tract- and county-level data from the 2020 ACS and imputed them as necessary (see Table A1).

Standardized values for the 17 measures were calculated with a mean of zero and an SD of 1. With standardized and unstandardized values, each measure was multiplied by its corresponding scoring coefficients and the raw score is equal to the sum of these products. These calculations are illustrated using values for each measure, measured at the minimum value of the 85^th^ percentile (the MSSP eligibility cutoff).

The 2 versions of the ADI—with and without standardization—were converted into percentiles to match the available Neighborhood Atlas data. The correlation between my percentiles using unstandardized values and the Neighborhood Atlas national percentiles is a measure of the fidelity between the Neighborhood Atlas approach and my specification of the ADI variables, imputation process, and choice of scoring coefficients. That is, did I get it right? Using both measures, for each state, I also calculated the number of block groups that had scores equal to or above the 85th percentile (the MSSP eligibility criteria). I then quantify the level of mismatch in eligibility across measures.

On July 10, 2023, after the exchange in *Health Affairs*, Neighborhood Atlas released an updated fourth version of the ADI. According to the change log,

the v4 ADI has minor standard shrinkage statistical updates included to mitigate the effect of year-to-year sampling variations in block group level component estimates within American Community Survey (ACS) data. This results in very little actual change in ADI ranking but buffers from known and future expected variation in ACS source data.^13^

This new version seems to address another major problem with the ADI, that of small sample sizes at the block group level and estimates that are quite imprecise (even in the ACS data pooling across 5 years).^22^ This problem is particularly acute for measures based on a subset of ACS households—for instance, median home value and median mortgage are only available for owner-occupied households. In block groups in the lowest decile of home ownership (with <22.8% owning), the mean margin of error for median home values is an astoundingly high $105 418 (see Table A6). In that same decile, despite a mean poverty rate of 21.0%, median home values are $349 701, well above the national average of $290 545. The “shrinkage” method may involve using information from other measures or from an earlier period or from different geographic levels to increase any given measure's precision.^23^ Exactly what was done and how many block groups have imputed values is not documented.

In the analysis of the relationship between home ownership and median home values reported in Table A6, there is only a minor change across versions in the national ranking of the lowest decile of home ownership. Whatever the case, shrinkage techniques have nothing to do with the problem of standardization. Where appropriate, I compared the 2 versions of the Neighborhood Atlas ADI to determine whether the new version would alter my main findings.

## Results

There was a total of 242 335 block groups in the United States, the District of Columbia, and Puerto Rico in the 2020 ACS, the same count obtained by Neighborhood Atlas. A total of 5982 block groups did not meet the Neighborhood Atlas's inclusion criteria using version 3.2 and 7036 did not meet the inclusion criteria using version 4. Even after imputation of tract- and county-level data, there was still a small number (*n* = 217) of block groups with missing information for income disparity, “no phone,” median rent, and/or median mortgage; dropping these block groups reduced the final sample size to 236 136. Descriptive statistics are reported in Table A2.

###  

#### Illustration of calculation of ADI scores

To illustrate the difference in the calculation of the ADI with nonstandardized or standardized values, I calculated scores at the 85th worst percentile for each of the 17 measures using both approaches (Table 1). The median home values, for example, in the worst 85th percentile was $96 800, which is an SD of −0.721 below the mean. Multiplying these unstandardized and standardized values by Singh's scoring coefficients for home values (−0.0688) yields a product of −6 659 for the unstandardized value and −0.05 for the standardized value. After repeating these calculations for each measure, the raw ADI scores were obtained by summing the products. The unstandardized score was −10 390 and the standardized value was 1.104.

**Table 1.** Calculation of ADI score at the 85th percentile using standardized and unstandardized values.

		Unstandardized ADI			Standardized ADI		
	Scoring coefficient	Value	Product	Percent	Value	Product	Percent
FPL 150%	0.1037	0.41	0.04	0.0%	1.003	0.10	9.4%
FPL 100%	0.0977	0.21	0.02	0.0%	0.800	0.08	7.1%
Income disparity^a^	0.0936	3.94	0.37	0.0%	0.963	0.09	8.2%
Less than 8 years of education	0.0849	0.10	0.01	0.0%	0.650	0.06	5.0%
12 or more years of education	−0.0970	0.77	−0.07	0.0%	−0.941	0.09	8.3%
White collar occupation	−0.0874	0.39	−0.03	0.0%	−1.056	0.09	8.4%
Unemployed	0.0806	0.11	0.01	0.0%	0.714	0.06	5.2%
Single parent	0.0719	0.27	0.02	0.0%	0.889	0.06	5.8%
Owner occupied	−0.0615	0.33	−0.02	0.0%	−1.198	0.07	6.7%
Crowding	0.0556	0.07	0.00	0.0%	0.567	0.03	2.9%
No motor vehicle	0.0694	0.16	0.01	0.0%	0.562	0.04	3.5%
No telephone	0.0877	0.04	0.00	0.0%	0.591	0.05	4.7%
No plumbing	0.0510	0.00	0.00	0.0%	0.000	0.00	0.0%
Median income ($)	−0.0977	36 854.00	(3600.64)	34.7%	−0.884	0.09	7.8%
Median home value ($)	−0.0688	96 800.00	(6659.84)	64.1%	−0.721	0.05	4.5%
Median rent ($)	−0.0781	692.00	(54.05)	0.5%	−0.882	0.07	6.2%
Median mortgage ($)	−0.0770	985.00	(75.84)	0.7%	−0.915	0.07	6.4%
Total (raw score)			(10 390.01)	100.0%		1.104	100.0%

Scoring coefficients are from Table 1 of Singh (2003).^12^ Values are minimum values at the 85th percentile for each measure, reverse coding values with a negative scoring coefficient. Data are from the 2020 American Community Survey, 5-year estimates, block group. “Product” is equal to the scoring coefficient multiplied by the measure's value. The “Percent” is equal to the product divided by the sum of the products.

Abbreviations: ADI, Area Deprivation Index; FPL, Federal Poverty Level.

^a^Income disparity is equal to the ln(household income <$10 000/household income >$50 000).

Dividing the product for each measure by the total score, I obtained the implicit weights using either nonstandardized or standardized values. Just 2 measures—median income and median home value—account for 98.8% (34.7% + 64.1%) of the unstandardized ADI score. The other 2 indicators measured in dollars—rent and mortgage—account for just 1.2% of the score. The remaining 13 measures combined contribute less than 0.01%. In Table A3, I show the distribution of the combined contribution of income and home value to the unstandardized score for all 236 136 block groups. The mean of the distribution is 98.98% (SD = 0.41%); 99% of block groups have a combined value greater than 97.49%.

In contrast to the unstandardized ADI, the standardized ADI is truly multidimensional, with all measures contributing to the score (Table 1). Percentages obtained by dividing each product by the total raw score roughly align with the scoring coefficients. The differences between the 2 may be due to the nonnormal distribution of most of these measures (see Table A1). The “No Plumbing” variable is so skewed that all households have plumbing at the 85th percentile (plumbing being nearly ubiquitous).

A related problem with nonstandardized values is that the relative contribution of income and housing is sensitive to the ratio of the 2. In block groups where houses are relatively affordable compared to incomes, a greater implied weight is assigned to income than housing and the reverse is true where houses are relatively less affordable. I calculated the ratio of median home value to median income and converted these ratios into deciles. For every decile, I used the above approach to calculate the contribution of house value and income to the raw ADI score (see Table A3). In the bottom decile with home values less than 1.75 greater than income, the contribution of income (49.7%) is roughly the same as the contribution of house value (48.9%). By contrast, in the top decile, with home values more than 7.32 greater than incomes, the contribution of income (11.5%) is dwarfed by the contribution of housing values (87.8%).

#### Comparison of different versions of the ADI

The standardized and unstandardized ADI scores were converted into percentiles to compare them with those publicly available from Neighborhood Atlas. Table 2 presents a correlation matrix of these 2 measures, the 2 versions of the Neighborhood Atlas ADI (v3.2 and v4.0), as well as percentile scores for median income and median home values. The percentiles for my unstandardized ADI are basically identical to the 2020 Neighborhood Atlas (v3.2) percentiles, with a correlation greater than 0.9999—just 5.5% of the percentiles do not match and the absolute value of the difference between the 2 measures is never greater than 1 percentile point. In short, I succeeded with considerable accuracy in reproducing the percentile scores available from the Neighborhood Atlas.

**Table 2.** Pairwise correlations of alternative versions of the ADI and select measures.

	Atlas ADI 3.2	Atlas ADI 4.0	ADI–not STD	ADI–STD	Home value	Income
Neighborhood Atlas ADI (3.2)	1.00000					
Neighborhood Atlas ADI (4.0)	0.94520	1.00000				
ADI–not STD	0.99997	0.94514	1.00000			
ADI–STD	0.72454	0.67961	0.72498	1.00000		
Median home value (percentile)	−0.98447	−0.93855	−0.98425	−0.64446	1.00000	
Median income (percentile)	−0.77136	−0.70611	−0.77203	−0.87526	0.66390	1.00000

The 2020 Neighborhood Atlas ADI, version 3.2 and version 4.0, national rank percentiles were downloaded from https://www.neighborhoodatlas.medicine.wisc.edu. Version of the ADI using nonstandardized (ADI-not STD) and standardized values (ADI-STD) were calculated by the author using 2020 American Community Survey block-group–level data. Except for version 4.0 of the Neighborhood Atlas ADI, all correlations are based on 236 136 block groups in the United States and Puerto Rico. This count drops to 235 082 using ADI version 4.0.

Abbreviations: ADI, Area Deprivation Index; STD, standardized.

The correlation between the 2 versions of the Neighborhood Atlas—v3.2 and v4.0—is also substantial (0.9452) but does suggest, contrary to the Neighborhood Atlas claim, that the new “shrinkage” methodology had a nonnegligible impact on rankings. Percentile scores across the versions changed for 89.4% of the block groups, with about 10% increasing their ranking by 10 percentile points or more and another 10% with a decrease in ranking of 10 percentile points or more.

As expected, there is a very strong association between the Neighborhood Atlas ADI (v3.2) and home values (−0.9845)—a correlation nearly identical to that obtained by Hannan and colleagues^9^ for New York State—and a weaker association with median income (−0.7714). The correlations of these 2 measures with v4.0 are a bit weaker: −0.93855 with median home values and −0.70611 with income. By contrast, the ADI using standardized values (ADI-STD) is more strongly correlated with income (*r* = −0.8752) than with home values (*r* = −0.6444). This makes sense because the ADI includes 4 different measures of income.

At risk of belaboring the obvious, I calculated a simple version of the ADI with just 2 measures—median income and median home values—and weighted them with the scoring coefficients from Singh,^12^ reported in Table 1:

SimpleADI=(0.0688*HomeValue)+(0.0977*Income)

The correlation between the Neighborhood Atlas ADI rankings (v3.2) and the percentile scores of this simple unstandardized ADI is equal to 0.999995. The simple ADI percentiles did not match the Neighborhood Atlas ADI for 7.2% of the block groups and were off by just 1 percentile for the remaining block groups, except for 1 block group off by 2 percentiles (see Figure A2, left “household income” panel). These results are almost as good as using all 17 measures (with a non-match rate of 5.5%). In other words, to recreate the Neighborhood Atlas ADI national rankings, it is sufficient calculate a weighted average of just 2 measures.

Another check on my approach is to repeat Singh PCA using 2020 ACS data.^12^ This also allows for a comparison of the standardized ADI created using tract-level scoring coefficients with 1990 data to a standardized ADI with scoring coefficients from an analysis of 2020 block-group data. Despite some substantive differences in the factor loadings and scoring coefficients, there is a 0.988 correlation between the 2 ADI measures using standardized values (Table A5). This shows that following Singh's approach rather the Neighborhood Atlas approach yields very similar factor scores (and percentiles) with data collected 30 years apart and at different geographic levels.

#### The District of Columbia

In their examination of the Neighborhood Atlas ADI, Azar and colleagues^8^ showed that there was a weak association between the ADI and life expectancy in the cities they examined. The root of the problem can be illustrated by a closer look at Washington, DC, using alternative versions of the ADI, as well as percentile scores for median home value, median income, and percent owner occupied.

**Figure 1.**
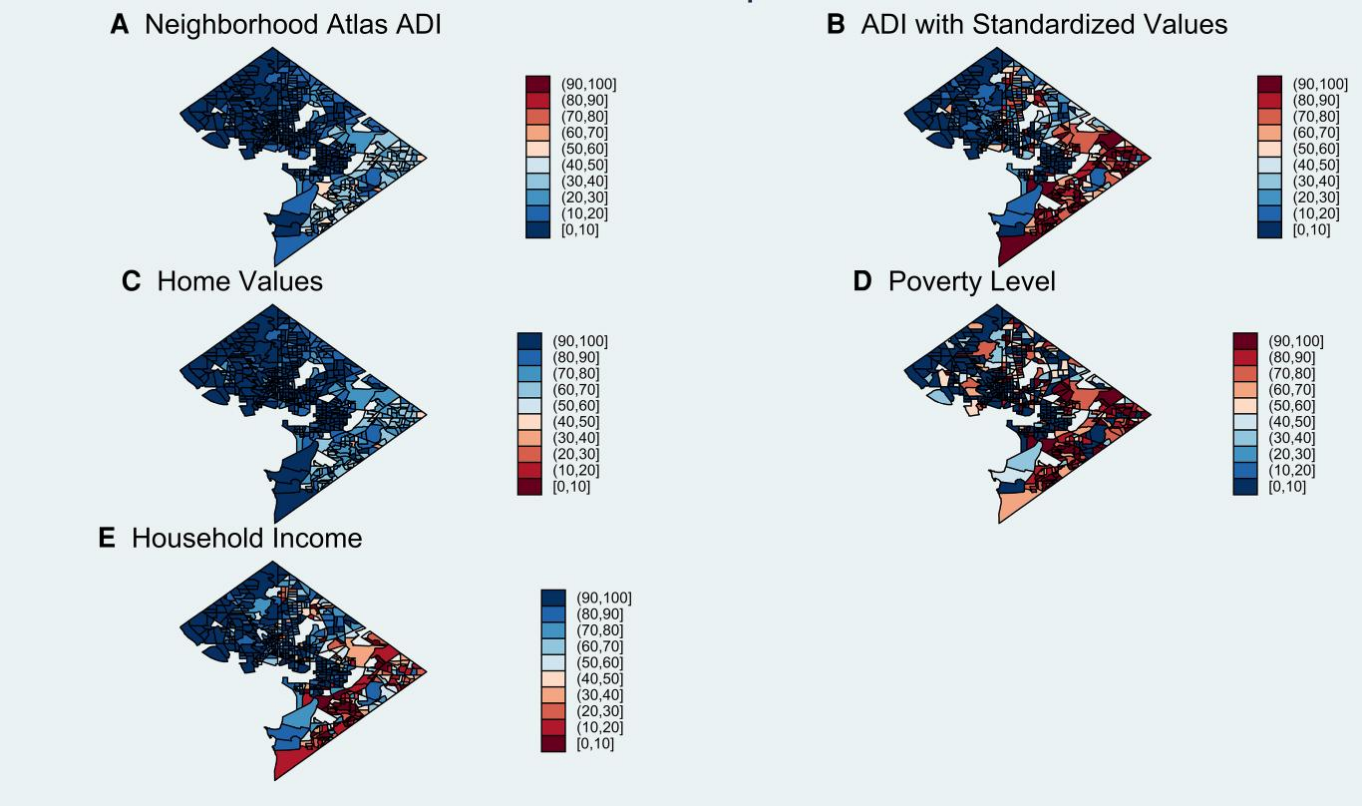
Alternative measures of area deprivation in the District of Columbia. Source: 2020 Neighborhood Atlas ADI (version 3.2, retrieved from https://www.neighborhoodatlas.medicine.wisc.edu). All other measures were based on the author's analysis of 2020 American Community Survey data. Home values are reverse coded. Abbreviation: ADI, Area Deprivation Index.

Mapping the data shows that nearly the entire District lies in the 3 most advantaged Neighborhood Atlas ADI deciles (scores <30) (Figure 1). The map of median home value percentiles is nearly identical to the Neighborhood Atlas ADI; the correlation between the Neighborhood Atlas ADI and home values in the District is −0.974. The map using the standardized ADI is much more colorful—resembling the maps using poverty percentiles (*r* = 0.737) and median income (*r* = −0.886)—and is a more accurate depiction of deprivation across the District.

#### Eligibility for the MSSP

Azar and colleagues^8^ found that no block group in Washington, DC, would be eligible for the MSSP using the Neighborhood Atlas ADI. By contrast, using the standardized ADI, I calculate that 85 of the 532 block groups, or 16%, would be eligible. I repeated this calculation for each state as well as Puerto Rico (see Table A4a). This is not just a problem of some big cities. In California as a whole, just 1.5% of its block groups would be eligible using the Neighborhood Atlas ADI compared to 16.0% using the standardized ADI. Other states would benefit. In Ohio, for example, 28.7% are eligible using the Neighborhood Atlas ADI compared to 18.3% using the standardized percentiles.

**Figure 2.**
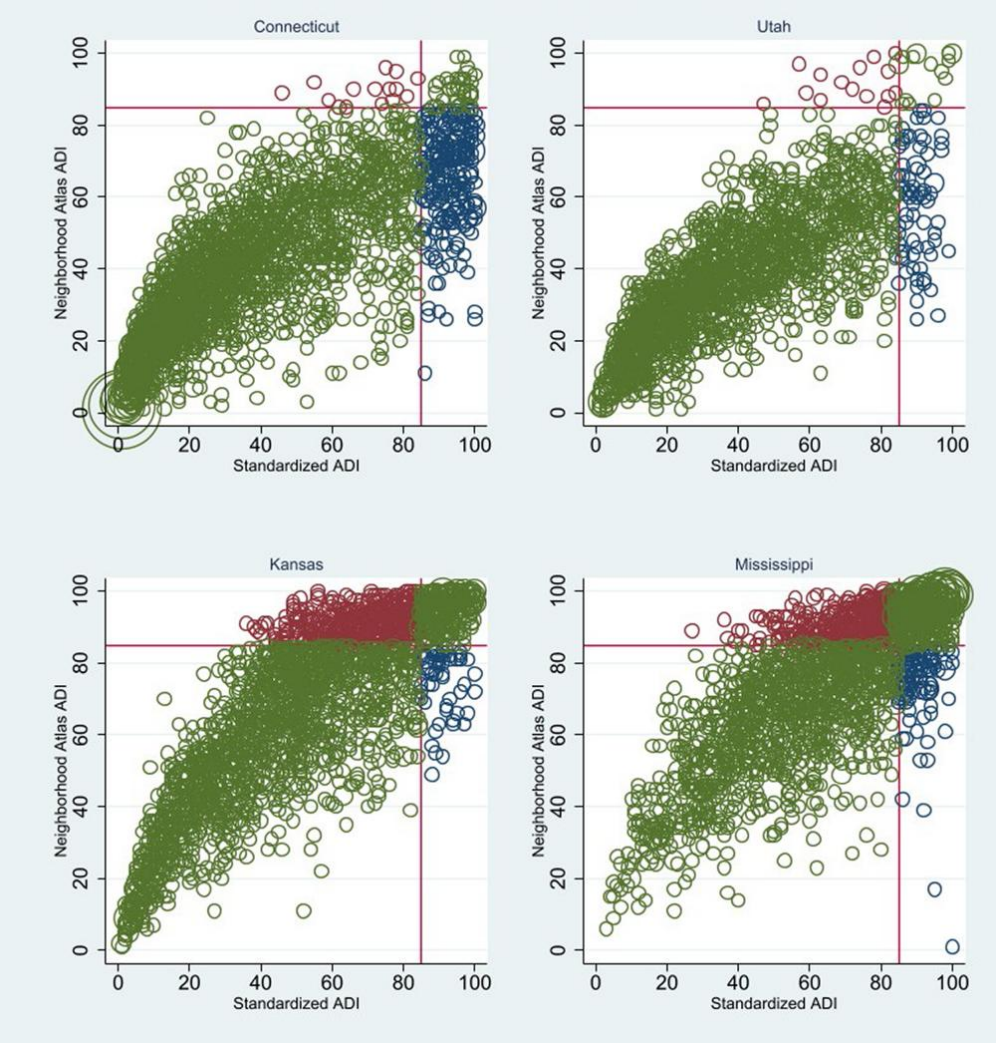
Differences in MSSP eligibility across ADI measures, select states. Source: 2020 Neighborhood Atlas ADI (version 3.2. retrieved from https://www.neighborhoodatlas.medicine.wisc.edu). Standardized ADI based on the author's analysis of 2020 American Community Survey data. Abbreviations: ADI, Area Deprivation Index; MSSP, Medicare Shared Savings Program.

Figure 2 shows scatterplots of the 2 versions of the ADI for 4 representative states. Block groups colored in blue are those that are eligible using the standardized ADI but not with the Neighborhood Atlas ADI; those colored in orange are eligible only using the unstandardized values. In Connecticut, of the 295 block groups eligible using the standardized ADI, just 36 were also eligible using the Neighborhood Atlas ADI (and just 14 were only eligible using the Neighborhood Atlas ADI, not the standardized ADI).

Kansas is a state that benefits considerably using the Neighborhood Atlas ADI, with 30% of the block groups having percentile scores at or above 85. This qualifies Kansas as the seventh most “deprived” state behind Mississippi, Arkansas, West Virginia, Alabama, Kentucky, and Oklahoma (see Tables A4a and A4b). Using the standardized ADI, by contrast, just 13.2% of block groups in Kansas have scores equal to or above 85, placing the state right in the middle of the distribution as the 27th most deprived state alongside Pennsylvania (26th) and Florida (28th). Totaling these values across all block groups nationwide (including Puerto Rico), just 20 477 had scores equal to or above the 85th percentile using both the standardized ADI and the Neighborhood Atlas ADI (Table A4b). Put differently, of the 37 778 blocks that are eligible using the standardized ADI, just 54.1% (= 20 447/37 778) are eligible using the Neighborhood Atlas ADI. This mismatch nationally is also evident in individual states. While Virginia as a whole fares roughly as well using either the standardized (8.9% eligible) or unstandardized (8.4%) ADI, within the state just 46.6% (= 240/514) of those eligible using the standardized version are also eligible using the unstandardized version (Table A4b).

## Discussion

The argument and evidence in this paper are straightforward. From a statistical point of view, it is not sound to use unstandardized values in computing scores after a factor analysis when the measures have very different scales. For the Neighborhood Atlas ADI, failure to standardize means that just 2 variables—median home value and median income—account for all the variation in scores. Obviously, if 2 measures account for more than 99% of a score, then the other 15 measures are irrelevant. While the Neighborhood Atlas ADI is marketed as a rich multidimensional deprivation index, the opposite is the case. This pattern is entirely consistent with the findings of critics of the Neighborhood Atlas ADI. If anything, they greatly underestimated the magnitude of the problem.

Failing to standardize also means that the weighting of these 2 measures varies substantially across block groups, with home values accounting for more than 85% of the raw score in places where home values are disproportionately greater than median incomes. This problem magnifies the inherent problem with the ACS median home value measure. It is not based on all homes, but only those occupied by owners. A handful of expensive homes in an otherwise disadvantaged neighborhood where nearly everyone rents (or such homes in adjacent block groups, when median home value is imputed from tract-level data) would make the block group's ranking substantially lower (see Tables A6 and A7). This problem is magnified by small sample sizes at the block-group level (even in the 5-year files).

In the response to their critics, Powell and colleagues^11^ argue that “true validation” lies in the large number of studies that have used the Neighborhood Atlas ADI showing an association with a range of health and health service use outcomes. This may be the case, strictly speaking, but almost any decent composite measure and many single indicators show similar associations with health outcomes, given the high correlation among different indicators of deprivation.^24,25^ The correlation between the standardized ADI and median income reported above is −0.87. Arguably, the single most widely used “validated” measure of area deprivation is not the ADI but the single-dimension area poverty rate.^26,27^

## Conclusion

This analysis compared 2 versions of the ADI, with and without standardization, holding everything else constant by using the Neighborhood Atlas methodology. As suggested in passing, the problems with the ADI extend far beyond the standardization issue. This includes using tract-level scoring coefficients from Singh's 2003 analysis, the restriction to block groups, imputing values from tract- and county-level data, and not weighting data to reflect differences in population sizes across block groups. Further analysis is necessary to determine if the ADI has any merit even if the standardization problem is addressed. This includes a re-examination the underlying data and measures, the factor structure, the imprecision of block-level values, and the thorny issue of median home values as a measure of deprivation. Researchers who have used the Neighborhood Atlas ADI over different years and versions to produce “nearly 1000 peer reviewed scientific articles” may want to check if the measure they used is simply a weighted average of median income and median home values. If so, does this alter the interpretation of their findings?

From a policy perspective, there are compelling reasons to reconsider the use of the Neighborhood Atlas ADI in determining eligibility for national payment programs such as the MSSP. The measure is not as advertised. It is based on 2 measures that are not, strictly speaking, measures of deprivation. It does a poor job of properly classifying block groups, especially in areas with relatively high and relatively low median home values. While Neighborhood Atlas researchers are dismissive of their critics showing that very disadvantaged areas in major cities would not be considered eligible, it seems unlikely that representatives of states such as Utah, California, and Connecticut would consider the Neighborhood Atlas ADI a fair measure.

## Supplementary Material

qxad063_Supplementary_Data
