# Supplementary material for: Deciphering the Neighborhood Atlas Area Deprivation Index: the consequences of not standardizing
Source: Health Aff Sch. 2023 Nov 3;1(5):qxad063. doi: 10.1093/haschl/qxad063 (PMC10986280; doi:10.1093/haschl/qxad063)
Supplement: qxad063_Supplementary_Data [file qxad063_Supplementary_Data.zip › Supplemental Material.docx]

**Supplemental Material (Appendix) for “Deciphering the Neighborhood Atlas Area Deprivation Index: The Consequences of Not Standardizing.”**

[Discussion of Analytic Methods 1](#_Toc148698481)

[ADI Measures 1](#_Toc148698482)

[American Community Survey Tables for ADI Measures 1](#_Toc148698483)

[Stata Code used to Create ADI Measures 2](#_Toc148698484)

[Discussion of Supplemental Tables 3](#_Toc148698485)

[References 7](#_Toc148698486)

[Supplemental Tables 8](#_Toc148698487)

[Table A1. Missing Values at Block Group Level, Before and After Neighborhood Atlas Exclusions 11](#_Toc148698488)

[Table A2. Descriptive Statistics 12](#_Toc148698489)

[Table A3. Contribution of Median Income and Median Home to Unstandardized Raw Score, by Ratio of Home Value to Income 13](#_Toc148698490)

[Figure A1. Median Home Value and Median Income Explain Almost All of the Variation in Unstandardized ADI Scores 14](#_Toc148698491)

[Table A4a.Classification of Block Groups using 85th Percentile Cutoff Using Alternative Versions of ADI 15](#_Toc148698492)

[Table A4b. Classification of Block Groups using 85th Percentile Cutoff Using Alternative Measures 16](#_Toc148698493)

[Table A5. Comparison of Factor Loadings and Scoring Coefficients, using 1990 and 2020 Data 17](#_Toc148698494)

[Table A6. Home Ownership, Home Values, Margins of Error and Missing Values 18](#_Toc148698495)

[Figure A2. Percentile Difference between Neighborhood Atlas ADI v32 and Simple ADI, using Alternative Median Income Measures 19](#_Toc148698496)

[Table A7. Select Characteristics of Least Deprived Decile using Neighborhood Atlas ADI, by Standardized ADI Deciles 20](#_Toc148698497)

## Discussion of Analytic Methods

### ADI Measures

Neither Singh nor Neighborhood Atlas indicate the source of the measures used in the construction of the ADI. To identify the tables, I relied on the information in a paper by Knighton and colleagues that used the Neighborhood Atlas method to calculate raw scores and percentiles for the state of Utah.^1^ Table 1 of this paper identifies the ACS tables they used. This table is also of interest because it contains an illustration of how to they used scoring coefficients and unstandardized values to obtain raw scores, as in Exhibit 1 of the main paper. For one block group in Utah, with their numbers it is easy to calculate that weighted values of median income and median home values account for 99.3% of the raw ADI score ([-14,021-3,922]/-18,073). Another interesting feature of this table is that they are using 2000 scoring coefficients obtained from Neighborhood Atlas researchers rather than 1990 scoring coefficients. I was unable to find any other mention in Neighborhood Atlas documentation of this (slightly) different set of scoring coefficients.

| American Community Survey Tables for ADI Measures | | | |
| --- | --- | --- | --- |
| Measure | Table | Table Name |  |
| 150% Federal Poverty Level | B17002 | Ratio Of Income to Poverty Level in The Past 12 Months |  |
| 100% Federal Poverty Level | C17010 | Poverty Status in The Past 12 Months of Families by Family Type by Presence of Related Children Under 18 Years |  |
| Income Disparity | B19001 | Household Income in The Past 12 Months (In Nominal Dollars) |  |
| Education, Less than 9 Years | B15003 | Educational Attainment for The Population 25 Years and Over |  |
| Education, 12 or More Years | B15003 | Educational Attainment for The Population 25 Years and Over |  |
| White Collar Occupation | C24010 | Sex By Occupation for The Civilian Employed Population 16 Years and Over |  |
| Unemployment Rate | B23025 | Employment Status for The Population 16 Years and Over |  |
| Single Parent Family | B11003 | Family Type by Presence and Age of Own Children Under 18 Years |  |
| Owner Occupied Household | B25003 | Tenure |  |
| Crowding | B25014 | Tenure By Occupants Per Room |  |
| No Car | B25044 | Tenure By Vehicles Available |  |
| No Plumbing | B25049 | Tenure By Plumbing Facilities |  |
| No Phone | B25043 | Tenure By Telephone Service Available by Age of Householder |  |
| Median Home Value | B25077 | Median Value (Dollars) |  |
| Median Income | B19013 | Median Household Income in The Past 12 Months (In Nominal Dollars) |  |
| Median Rent | B25064 | Median Gross Rent (Dollars) |  |
| Median Mortgage | B25088 | Median Selected Monthly Owner Costs (Dollars) By Mortgage Status |  |

While the Neighborhood Atlas documentation as well as Singh state they are using median *family* income, I used median *household* income. In preliminary work, I examined both measures and found that using household income substantially improves the match between my version of an unstandardized ADI and the Neighborhood Atlas ADI (v.32). This difference in matches using both measures is visualized in Exhibit A8 below.

| Stata Code used to Create ADI Measures | |
| --- | --- |
|  | gen f_150fpl=(c17002_002e+c17002_003e+c17002_004e+c17002_005e)/c17002_001e |
|  | gen f_poor=b17010_002e/b17010_001e |
|  | egen income_50000plus=rowtotal( b19001_011e-b19001_017e) |
|  | gen income_0_10000=b19001_002e |
|  | gen f_disparity=ln(100*(income_0_10000/income_50000plus)) |
|  | egen schoolle8=rowtotal(b15003_002e-b15003_012e) |
|  | egen schoolge12=rowtotal(b15003_017e-b15003_025e) |
|  | gen f_schoolle8=schoolle8/b15003_001e |
|  | gen f_schoolge12=schoolge12/b15003_001e |
|  | gen f_occupation=(c24010_003+c24010_039+c24010_027+c24010_063)/c24010_001 |
|  | gen f_unemp=b23025_005e/b23025_002e |
|  | gen f_singleparent=(b11003_010e+ b11003_016e)/b11003_001e |
|  | gen f_rent_occup =(b25003_001e-b25003_003e)/b25003_001e |
|  | gen f_crowding=(b25014_005e+ b25014_006e+ b25014_007e+ b25014_011e+ b25014_012e+ ///  b25014_013e)/b25014_001e |
|  | gen f_nocar=(b25044_003e+ b25044_010e)/b25044_001e |
|  | gen f_noplumbing=(b25049_004e + b25049_007e)/b25049_001e |
|  | gen f_nophone=(b25043_007e+b25043_016e)/b25043_001e |
|  | gen median_income=b19013_001e |
|  | gen median_house=b25077_001e |
|  | gen median_rent=b25064_001e |
|  | gen median_mortgage=b25088_002e |
|  | |

## Discussion of Supplemental Tables

[Supplemental Table A1](#_Table_A1._Missing) shows that many of the measures used in the construction of the ADI have missing values. The biggest problem is with two measures 1) the income disparity measure which could not be calculated for 27.58% of block groups because there were no households with incomes less than $10,000, and 2) median rent is missing for 25.15% of block groups because there are no renters in the block group. There are also missing value problems with the other three indicators measured in dollars. Median home value was missing for 7.62% of cases perhaps because there were no owner-occupied households in these block groups.

In the Neighborhood Atlas documentation,^2^ it is noted that “geographic imputation methods have been applied to address missing data in the key component areas. This nested geographic imputation uses census tract and county level data …” I assume this means that they substituted tract or, if necessary, county values for missing values at the block group level. To use this imputation method, I retrieved tract- and county-level data from the 2020 ACS and imputed them as necessary.

[**Supplemental Table A2**](#_Table_A2._Descriptive) displays descriptive statistics for the 17 ADI measures. A cursory examination of these measures suggests that several measures, while appropriate in 1990, should not be included in a contemporary index of deprivation. For example, even at the 75^th^ percentile, all households have indoor plumbing. The income disparity measure does not appear to measure disparity so much as extreme poverty—very few households have incomes below $10,000 but many have incomes above $50,000. This is an example of a measure (along with the "less than 9 years of education”) that Singh would update to reflect changes across time in income and education. The Neighborhood Atlas retains thresholds that were appropriate in 1990 but not now. The “No Phone” measure was replaced with a “No Internet” measure starting in 2020. No phone is retained because the Neighborhood Atlas documentation does not include a scoring coefficient for the new internet measure. Finally, the four measures measured in dollars are all skewed, suggested that they should be normalized.

[**Supplemental Table A3**](#_Table_A3._) shows the association between deciles of the ratio of median home value to median income and the relative contribution of home values and income to the raw score (as illustrated in Table 1 in the main paper). These results show that as the ratio of home value to income increases, home value contribute a greater amount to the overall raw score. In the lowest decile, where home values are less than 1.78 times median income, the contribution of median income and median home values are roughly the same (49.2% and 49.6%). In the top decline, with a ratio greater than 7.37, median home values account for 87.9% of the raw score.

[**Supplemental Figure A1**](#_Figure_A1._Median) displays a histogram of the combined contribution of median home value and median income to calculation of the raw score, as illustrated in Exhibit 1 in the main paper. across all block groups, median income and median home value basically account for most of the calculated raw scores. These two variables alone account for 99% of the raw ADI score, with little variation across block groups (SD=0.41%). The problems identified by other critics of the ADI are not an isolated phenomenon true for only big cities with expensive houses but true for all block groups. This figure also shows that all the other measures in the ADI have a negligible impact on the ADI score.

[**Supplementa**l **Table A4a**](#_Table_A4a.Classification_of) shows for every state, the District of Columbia (DC) and Puerto Rico, the number and percent of block groups that are eligible at the 85^th^ percentile using the Neighborhood Atlas ADI (v.32) and the ADI calculated using standardized values. It also shows the percent eligible using ADI (v.4.0). States are sorted by the difference in percent eligible using the standardized ADI and the Neighborhood Atlas ADI (version 3.2). This shows that the difference between the two is greatest in DC, however California is a close second: Just 1.5% of block groups in California are eligible using version 3.2 and 1.3% using version 4.0 substantially lower than the much more realistic 15.5% using the standardized ADI. Despite a ten-fold difference in the number of block groups in California (n=25,231) compared to Kansas (n=2,382), using the Neighborhood Atlas ADI there are nearly twice as many eligible block groups in Kansas (n=723) than in California (n=381).

Repeating this analysis using version 4.0 of the Neighborhood Atlas ADI does not markedly change the results. Indeed, the correlation between the two versions across the states of the measure of eligibility is equal to 0.998. In the DC, just 3 block groups are eligible using v4.0 (up from zero using v3.2). If anything, the percent of block groups eligible appears to increase the most in the states that benefiting the most from using the Neighborhood Atlas ADI. In Kansas, for instance, the percent eligible increased from 30.4% with v3.2 to 31.9% with v4.0; similarly, in Mississippi, eligibility increased from 45.5% to 49.6%.

[**Supplemental** **Table A4b**](#_Table_A4b._Classification) breaks the data down further showing the level of agreement in eligibility using Neighborhood Atlas (v3.2) ADI and a standardized ADI, corresponding to the four areas shown in the scatterplots Exhibit 4 in the main paper. For instance, of the 3,936 block groups in California in the 85^th^ percentile or higher using the standardized ADI, just 262 were also eligible using the Neighborhood Atlas ADI. Only 119 block groups were eligible using the Neighborhood Atlas ADI but not the standardized ADI. By contrast, in Kansas, 472 block groups were eligible using the Neighborhood Atlas ADI but not the standardized ADI.

**[Supplemental Table A5](#_Table_A5._Comparison)** shows factor loadings and scoring coefficients from Singh’s original 2003 analysis^3^ and those obtained using 2020 data. As noted in the main paper, an oddity of the Neighborhood Atlas methodology is the use of Singh’s scoring coefficients from a tract-level analysis using block group data from the 1990 Decennial Census. With the available 2020 ACS data, this limitation is overcome by repeating Singh’s analysis aligning years and geographies.

The Stata code for this factor analysis is straightforward and presumably like Singh’s original analysis, using just the *factor* and *predict* commands:

local var "f_150fpl f_poor f_disparity f_schoolle8 f_schoolge12 f_occupation f_unemp f_singleparent f_rent_occup f_crowding f_nocar f_nophone f_noplumbing median_income median_house median_rent median_mortgage"

factor `var', factor(1)

predict adi_2020

The 2020 factor loadings and scoring coefficients reported in Exhibit A6 are part of the Stata output. The scoring coefficients can also be produced by regressing the *standardized value* of each measure on the predicted raw score, as follows:

foreach var in `var' {

egen s_`var'=std(`var')

}

regress adi_2020 s_`var’

While factor loadings reported by Singh are all greater than 0.40 (his criteria for including the indicator in his model), 5 of the 17 loadings using 2020 data are less than 0.40. Notably, there is a weak association between the ADI and common markers of deprivation in other indices, including unemployment, crowding, renting and no car. Comparing the tract scoring coefficients reported by Singh with those obtained at the block group level using 2020 ACS data, it is first important to note that the magnitude of the 1990 and 2020 scoring coefficients are sufficiently similar, indicated that however Singh obtained his scoring coefficients, they are based on a standardization of all measures.

There are also several noteworthy differences in scoring coefficients between 1990 to 2020. For instance, the unemployment score dropped from .081 to 0.23 and percent without a telephone dropped from .051 to 0.019. At the same time the absolute value of the income-related measures increased across the board; notably, the scoring coefficient of median income nearly doubled in size from 0.0977 to 0.181. Together the sum of the absolute values of the four income measures—FPL 150%, FPL 100%, income disparity and median income—increased from 0.393 to 0.604.

After converting the 2020 ADI scores into percentiles, I obtained the following pairwise correlation matrix:

|  | ADI-2020 | ADI-STD (1990) | ADI-V4 | ADI-V32 |
| --- | --- | --- | --- | --- |
| ADI2020 | 1.0000 |  |  |  |
| ADI-STD (1990) | 0.9881 | 1.0000 |  |  |
| ADI-V4 | 0.7042 | 0.6800 | 1.0000 |  |
| ADI-V32 | 0.7520 | 0.7244 | 0.9452 | 1.0000 |
| *ADI-STD (1990)* are percentiles for the standardized ADI score using 1990 scoring coefficients; ADI-V4 and ADI-V32 are the two versions of the ADI national rankings obtained from the Neighborhood Atlas website. | | | | |

Despite all the differences across factor loadings and scoring coefficients, the correlation between the standardized ADI percentile scores calculated using 1990 scoring coefficients and percentile scores obtained with 2020 data was 0.988. By contrast, the correlations of the Neighborhood Atlas national rankings are much lower (0.7038 with v4 and 0.7516 with v32).

[**Supplemental Table A6**](#_Table_A6._Home) examines median home values and ADI rankings across deciles of home ownership. We show estimates of median home values and margins of error (MOE) as reported in the ACS as well as values after imputing data from higher levels of geography. There are several noteworthy findings. First, in the lowest decile (with ownership rates below 23.8%), median home values are missing for nearly half of the block groups. Second, also in the first decile, the margin of error—a measure of precision—is quite high ($103,858). Third, across the ten ownership deciles, there is a U-shaped relationship between median home values, with higher values in the lowest and highest deciles. Fourth, there is a relatively weak relationship between home ownership and Neighborhood Atlas scores. Fifth, while version 4.0 of the ADI was meant to address the imprecision of estimates with “standard shrinkage” techniques, these changes had little impact on the mean rankings across ownership deciles. Finally, as expected there is a strong association between home ownership and poverty, with more than a six-fold difference between the top (3.3% poor) and bottom decile (21.0%).

[Supplemental Table A7](#_Table_A7._) shows another way of illustrating the problem caused by home values based on a small number of homeowners. The analysis is restricted to block groups in the bottom, least deprived, decile of the Neighborhood Atlas ADI, presenting summary measures for the ten deciles of the standardized ADI. There were 23,639 block groups in the lowest Neighborhood Atlas ADI decile, of these just 14,205 also in the lowest standardized ADI. Across the ten deciles, home values are quite high, with the highest value in the first decile ($953,572) and the second highest in the 10^th^ decile ($934,997), which is otherwise quite disadvantaged, with a median income of $33,427, a poverty rate of 35.5% and a mean standardized ADI percentile score of 95.3. A likely explanation of this is that just 8.9% of homes in this decile are owner occupied.

[**Supplemental Figure A2**](#_Figure_A2._Percentile) shows the difference between the Neighborhood Atlas percentiles and the percentiles of simple ADI calculated as a weighted average of median income and median home values (see above). The purpose of this figure is to demonstrate that while the Neighborhood Atlas claim they are using family income,^4,5^ they in fact are using household income. In the ACS, family income and household income are substantively different, given the way households and families are defined.^6^ Households consist of all people who occupy a housing unit, including related family members and all unrelated people. Families, by contrast, are groups of two people or more related by birth, marriage, or adoption residing together. Median family income are substantially higher than median household incomes because it exclude include non-family households (mainly single persons) who have generally have lower incomes than families.

There are two noteworthy findings. First, the household income figure shows the quality of the match between a simple ADI and the Neighborhood Atlas ADI (v3.2). The percentiles are the same 92.8% of the time and the difference for the remaining block groups is either -1 or +1 except for just one block group (with a difference of -2). This distribution is nearly identical to that of the difference between the standardized ADI using all 17 measures and the Neighborhood Atlas ADI (v3.2). Second, using family income instead of household income substantially decreases the agreement between the Neighborhood Atlas ADI (v.32) and the simple ADI. There is a perfect match for just 17.0% of the block groups and another 25% are off by 1 percentile. There are long tails on either end of the distribution, with 5.8% of block groups with a difference of -5 or lower and another 10.3% with a difference of 5 percentiles or greater. The only plausible explanation of the difference in the agreement using either family or household income is that, despite their available documentation, the Neighborhood Atlas used median household income (from ACS Table B19013) rather than median family income (from Table B19113).

## References

1. Knighton A, Savitz L, Belnap T, Stephenson B, VanDerslice J. Introduction of an Area Deprivation Index Measuring Patient Socio-economic Status in an Integrated Health System: Implications for Population Health. *EGEMs Gener Evid Methods Improve Patient Outcomes*. 2016;4(3). doi:10.13063/2327-9214.1238

2. Center for Health Disparities Research, University of Wisconsin School of, Medicine and Public Health. Neighborhood Atlas change log. Published July 2023. Accessed July 29, 2023. https://www.neighborhoodatlas.medicine.wisc.edu/changelog

3. Singh GK. Area Deprivation and Widening Inequalities in US Mortality, 1969–1998. *Am J Public Health*. 2003;93(7):1137-1143.

4. Kind AJH, Jencks S, Brock J, et al. Neighborhood socioeconomic disadvantage and 30-day rehospitalization: a retrospective cohort study. *Ann Intern Med*. 2014;161(11):765-774. doi:10.7326/M13-2946

5. University of Wisconsin School of Medicine and Public Health. 2020 Area Deprivation Index, v.3.2. Accessed July 15, 2023. https://www.neighborhoodatlas.medicine.wisc.edu

6. US Census Bureau. Subject Definitions. Census.gov. Published ND. Accessed September 8, 2023. https://www.census.gov/programs-surveys/cps/technical-documentation/subject-definitions.html

## Supplemental Tables

Table A1

Caption: Missing Values at Block Group Level, Before and After Neighborhood Atlas Exclusions

Source: Author’s analysis of American Community Survey, 2020.

Notes: ACS- American Community Survey; ADI - Area Deprivation Index. Income disparity is equal to the ln(household income <$10,000/household income>$50,000). Before calculating percentiles, the Neighborhood Atlas excludes 5,982 block groups due to 1) low/zero population or households, 2) large concentration of groups quarter and c) implausible values.

Table A2

Caption: Descriptive Statistics

Source: Author’s analysis of American Community Survey, 2020.

Notes: Income disparity was defined as the log of 100 x the ratio of number of households with < $10 000 income to number of households with ≥$50 000 income. Calculations are based on 236,136 block groups that a) meet the inclusion criteria of the Neighborhood Atlas ADI, and 2) do not have missing values after imputations.

Table A3

Caption: Contribution of Median Income and Median Home to Unstandardized Raw Score, by Ratio of Home Value to Income

Source: Author’s analysis of American Community Survey, 2020.

Notes: The contributions of each measure were obtained using the approach outlined in Exhibit 1. That is, the contribution of a measure is equal to the product of its scoring coefficient and the unstandardized value divided by the total raw score. The Total column is sum of the contributions of home value and income.

Figure A1

Caption: Median Home Value and Median Income Explain Almost All of the Variation in Unstandardized ADI Scores

Source: Author’s analysis of American Community Survey, 2020.

Notes: The combined contribution of median home values and median income are obtained for each block group by multiplying the unstandardized values of each of the two measures by their corresponding scoring coefficients. Then, these two products were summed and divided by the ADI raw score.

Table A4

Caption: Classification of Block Groups using 85th Alternative Versions of ADI, a. Marginal Counts and Percent and b. Detailed Classification

Source: 2020 Neighborhood Atlas ADI (v 3.2) retrieved from https://www.neighborhoodatlas.medicine.wisc.edu. Standardized ADI based on author's analysis of 2020 American Community Survey data.

Notes: Counts for Atlas ADI v4.0 are not shown. Across states, the correlation between the Percent Eligible ADI 4.0 and Percent Eligible 3.2 is .998; the correlation between Percent Eligible Std and Atlas 3.2 is 0.799.

Table A5

Caption: Comparison of Factor Loadings and Scoring Coefficients, using 1990 and 2020 Data

Source: The 1990 factor loadings and scoring coefficients are from Singh (2003, Table 1). The 2020 estimates are from author's analysis of the 2020 American Community Survey.

Note: Singh's analysis is at the Census tract-level, the analysis of the 2020 is at the block group level. Income disparity is equal to the ln(median income<$10,000/median income>$50,000).

Table A6

Caption: Home Ownership, Home Values, Margins of Error and Missing Values

Source: The 2020 Neighborhood Atlas ADI, version 3.2 and version 4.0 national rank percentiles were downloaded from https://www.neighborhoodatlas.medicine.wisc.edu. All other estimates were calculated by author using 2020 ACS block level data.

Notes: Except for Version 4.0 of the Neighborhood Atlas ADI, all results are based on all block groups in the U.S., DC and Puerto Rico with valid ADI v32 rankings. MOE-Margin of Error. Imputed-Median home value after imputation from tract- and county-level.

Table A7

Caption: Table A7. Select Characteristics of Least Deprived Decile using Neighborhood Atlas ADI, by Standardized ADI Deciles

Source: The 2020 Neighborhood Atlas ADI, version 3.2 national rank percentiles were downloaded from https://www.neighborhoodatlas.medicine.wisc.edu. All other estimates were calculated by author using 2020 ACS block level data.

Notes: Except for Version 4.0 of the Neighborhood Atlas ADI, all results are based on all block groups in the U.S., DC and Puerto Rico with valid ADI v32 rankings. MOE-Margin of Error. Imputed-Median home value after imputation from tract- and county-level.

Figure A2

Caption: Percentile Difference between Neighborhood Atlas ADI v32 and Simple ADI, Using Alternative Median Income Measures

Source: The 2020 Neighborhood Atlas ADI, version 3.2 national rank percentiles were downloaded from https://www.neighborhoodatlas.medicine.wisc.edu. All other estimates were calculated by author using 2020 ACS block level data.

| Table A1. Missing Values at Block Group Level, Before and After Neighborhood Atlas Exclusions | |  |
| --- | --- | --- |
|  | | |
|  | **Percent Missing** | |
| **Measure** | **ACS** | **After ADI exclusions^b^** |
| Population below 150% of the poverty threshold, % | 1.13 | 0.00 |
| Families below poverty level, % | 1.18 | 0.00 |
| Income disparity^a^ | 28.55 | 27.58 |
| Population aged ≥ 25 y with < 9 y of education, % | 1.18 | 0.00 |
| Population aged ≥ 25 y with at least a high school diploma, % | 1.94 | 0.77 |
| Employed persons aged ≥ 16 y in white-collar occupations, % | 1.18 | 0.00 |
| Civilian labor force population aged ≥16 y unemployed, % | 1.10 | 0.00 |
| Single-parent households with children aged < 18 y, % | 1.35 | 0.07 |
| Owner-occupied housing units, % (home ownership rate) | 1.18 | 0.00 |
| Households with more than 1 person per room, % (crowding) | 0.96 | 0.00 |
| Households without a motor vehicle, % | 0.96 | 0.00 |
| Households without a telephone, % | 1.35 | 0.07 |
| Occupied housing units without complete plumbing, % | 1.08 | 0.00 |
| Median household income, $ | 9.16 | 7.62 |
| Median home value, $ | 6.48 | 5.11 |
| Median gross rent, $ | 12.44 | 10.88 |
| Median monthly mortgage, $ | 26.12 | 25.15 |
| Block Groups | 242,339 | 236,353 |
| Source: Author’s analysis of American Community Survey, 2020. | | |
| Notes: ACS- American Community Survey; ADI - Area Deprivation Index |  |  |
| a Income disparity is equal to the ln(household income<$10,000/household income>$50,000) | | |
| ^b^ Before calculating percentiles, the Neighborhood Atlas excludes 5,982 block groups due to 1) low/zero population or households, 2) large concentration of groups quarter and c) implausible values. | | |

|  |  |  |
| --- | --- | --- |

| Table A2. Descriptive Statistics | | | |  |  |
| --- | --- | --- | --- | --- | --- |
|  |  |  | **Percentile** | | |
| **Measure** | **Mean** |  | **50th** | **25th** | **75th** |
| Population below 150% of the poverty threshold, % | 22.5% |  | 17.8% | 8.2% | 32.4% |
| Families below poverty level, % | 10.5% |  | 5.5% | 0.0% | 14.9% |
| Income disparity | 2.18 |  | 2.09 | 1.31 | 2.96 |
| Population aged ≥ 25 y with < 9 y of education, % | 5.1% |  | 2.3% | 0.0% | 6.5% |
| Population aged ≥ 25 y with at least a high school diploma, % | 88.0% |  | 91.7% | 83.2% | 96.6% |
| Employed persons aged ≥ 16 y in white-collar occupations, % | 59.0% |  | 59.0% | 45.9% | 72.8% |
| Civilian labor force population aged ≥16 y unemployed, % | 5.7% |  | 3.8% | 1.1% | 7.8% |
| Single-parent households with children aged < 18 y, % | 14.0% |  | 10.0% | 3.1% | 20.5% |
| Owner-occupied housing units, % | 65.4% |  | 72.1% | 48.0% | 87.2% |
| Households with more than 1 person per room, % | 3.4% |  | 0.0% | 0.0% | 4.2% |
| Households without a motor vehicle, % | 8.6% |  | 3.6% | 0.0% | 10.6% |
| Households without a telephone, % | 1.7% |  | 0.0% | 0.0% | 2.3% |
| Occupied housing units without complete plumbing, % | 0.4% |  | 0.0% | 0.0% | 0.0% |
| Median household income, $ | 72,327 |  | 63,144 | 44,813 | 89,694 |
| Median home value, $ | 290,550 |  | 207,000 | 127,000 | 355,500 |
| Median gross rent, $ | 1,188 |  | 1,057 | 790 | 1,446 |
| Median monthly mortgage, $ | 1,708 |  | 1,487 | 1,125 | 2,106 |
| Data: 2020 American Community Survey, 5-Year, Block Group Level. | | | | | |
| Income disparity is equal to the ln(household income<$10,000/household income>$50,000) | | | | | |
| Note: Calculations are based on 236,118 block groups that a) meet the inclusion criteria of the Neighborhood Atlas ADI, and 2) do not have missing values after imputations. | | | | | |

|  | | | | |  |
| --- | --- | --- | --- | --- | --- |
| Table A3. Contribution of Median Income and Median Home to Unstandardized Raw Score, by Ratio of Home Value to Income | | | | | |
| **Ratio of Home Value to Income** | |  | **Contribution to Unstandardized ADI Raw Score** | | |
| **Decile** | **Range** |  | **Income (%)** | **Home Value (%)** | **Total (%)** |
| 1 | <1.78 |  | 49.2 | 49.6 | 98.8 |
| 2 | 1.78-2.16 |  | 41.3 | 57.6 | 99.0 |
| 3 | 2.16-2.48 |  | 37.6 | 61.4 | 99.0 |
| 4 | 2.48-2.81 |  | 34.6 | 64.4 | 99.1 |
| 5 | 2.81-3.18 |  | 31.9 | 67.2 | 99.1 |
| 6 | 3.18-3.65 |  | 29.2 | 70.0 | 99.1 |
| 7 | 3.65-4.29 |  | 26.2 | 72.9 | 99.2 |
| 8 | 4.29-5.31 |  | 22.8 | 76.4 | 99.2 |
| 9 | 5.31-7.37 |  | 18.5 | 80.7 | 99.3 |
| 10 | >7.37 |  | 11.6 | 87.9 | 99.4 |
| Source: Author’s analysis of American Community Survey, 2020. | | | | | |
| Note: The contributions of each measure were obtained using the approach outlined in Exhibit 1. That is, the contribution of a measure is equal to the product of its scoring coefficient and the unstandardized value divided by the total raw score. The Total column is sum of the contributions of home value and income. | | | | | |

### Figure A1. Median Home Value and Median Income Explain Almost All of the Variation in Unstandardized ADI Scores


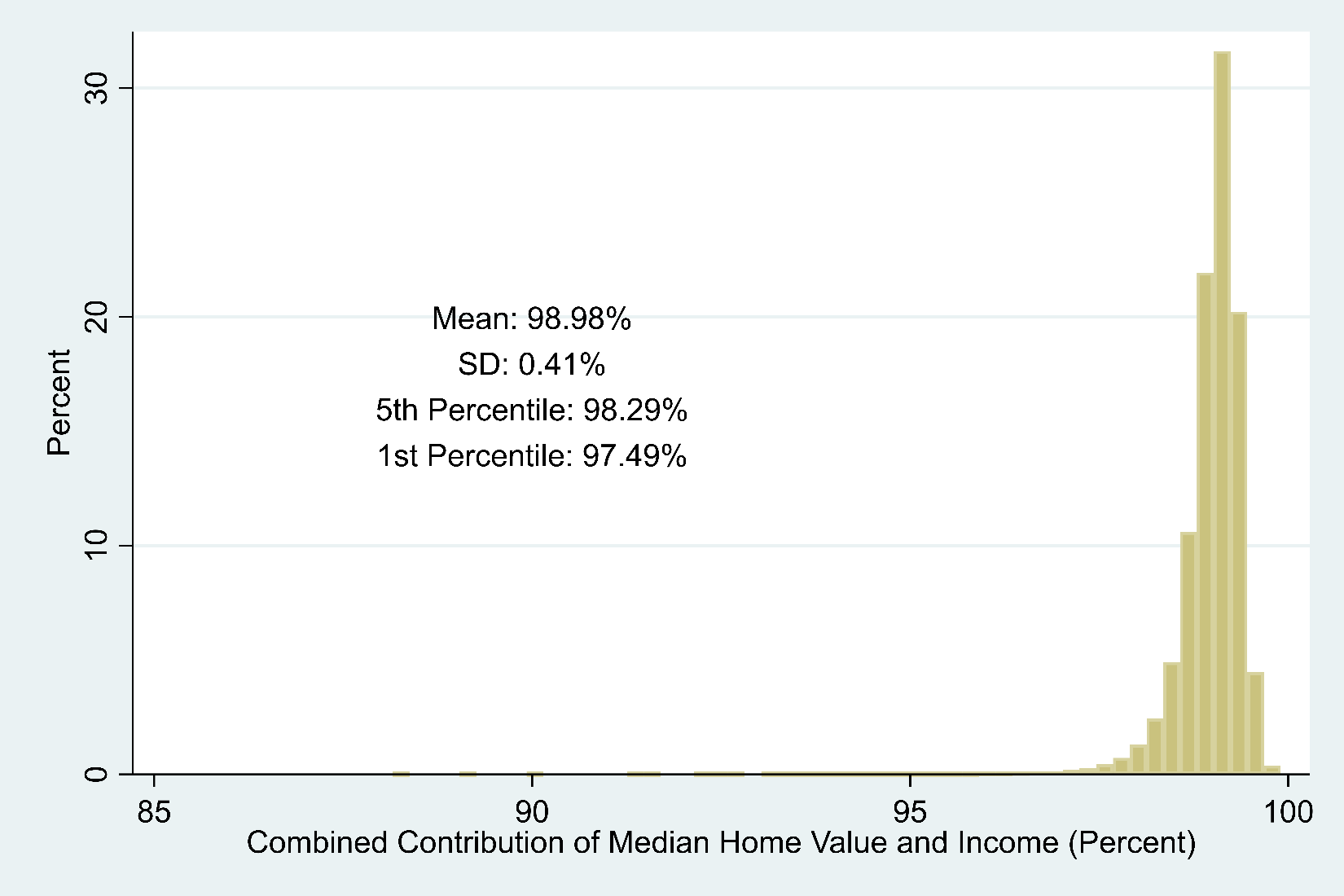


Source: Author's analysis of American Community Survey, 2020.

Notes: The combined contribution of median home values and median income are obtained for each block group by multiplying the unstandardized values of each of the two measures by their corresponding scoring coefficients. Then, these two products were summed and divided by the ADI raw score.

| Table A4a.Classification of Block Groups using 85th Percentile Cutoff Using Alternative Versions of ADI | | | | | | | |
| --- | --- | --- | --- | --- | --- | --- | --- |
| **a. Marginal Counts and Percents** | | | | | | | |
|  | **Counts** | | |  | **Percent ≥85** | | |
|  | **Total** | **Standardized ADI ≥85** | **Atlas ADI ≥85** |  | **Standardized ADI** | **Atlas ADI, V3.2** | **Atlas ADI, V4.0** |
| Sum | 236,118 | 37,778 | 37,779 |  | 16.0 | 16.0 | 16.0 |
| DC | 532 | 89 | - |  | 16.7 | 0.0 | 0.6 |
| CA | 25,231 | 3,910 | 381 |  | 15.5 | 1.5 | 1.3 |
| NV | 1,917 | 398 | 149 |  | 20.8 | 7.8 | 7.6 |
| RI | 767 | 92 | 7 |  | 12.0 | 0.9 | 1.0 |
| MA | 4,961 | 484 | 28 |  | 9.8 | 0.6 | 0.6 |
| CT | 2,660 | 293 | 50 |  | 11.0 | 1.9 | 1.7 |
| PR | 2,466 | 1,946 | 1,743 |  | 78.9 | 70.7 | 77.4 |
| NJ | 6,437 | 688 | 165 |  | 10.7 | 2.6 | 2.4 |
| NY | 15,300 | 2,430 | 1,250 |  | 15.9 | 8.2 | 9.1 |
| AZ | 4,656 | 882 | 579 |  | 18.9 | 12.4 | 11.9 |
| AK | 488 | 59 | 30 |  | 12.1 | 6.1 | 5.8 |
| OR | 2,924 | 234 | 63 |  | 8.0 | 2.2 | 1.6 |
| WA | 5,226 | 336 | 74 |  | 6.4 | 1.4 | 1.1 |
| HI | 1,011 | 51 | 2 |  | 5.0 | 0.2 | 0.7 |
| DE | 685 | 58 | 32 |  | 8.5 | 4.7 | 3.7 |
| MD | 3,982 | 363 | 222 |  | 9.1 | 5.6 | 5.6 |
| ID | 1,259 | 100 | 60 |  | 7.9 | 4.8 | 3.9 |
| UT | 1,979 | 91 | 32 |  | 4.6 | 1.6 | 1.5 |
| CO | 3,964 | 251 | 136 |  | 6.3 | 3.4 | 3.4 |
| NM | 1,570 | 432 | 394 |  | 27.5 | 25.1 | 26.2 |
| NH | 981 | 39 | 18 |  | 4.0 | 1.8 | 1.9 |
| MN | 4,569 | 272 | 213 |  | 6.0 | 4.7 | 3.4 |
| TX | 18,230 | 4,331 | 4,098 |  | 23.8 | 22.5 | 23.6 |
| VT | 535 | 14 | 8 |  | 2.6 | 1.5 | 0.6 |
| FL | 13,043 | 1,814 | 1,688 |  | 13.9 | 12.9 | 12.9 |
| LA | 4,180 | 1,139 | 1,100 |  | 27.2 | 26.3 | 27.5 |
| VA | 5,783 | 514 | 490 |  | 8.9 | 8.5 | 9.3 |
| WY | 450 | 17 | 19 |  | 3.8 | 4.2 | 3.4 |
| MT | 887 | 59 | 67 |  | 6.7 | 7.6 | 6.0 |
| GA | 7,310 | 1,370 | 1,458 |  | 18.7 | 19.9 | 21.6 |
| WI | 4,612 | 486 | 584 |  | 10.5 | 12.7 | 11.6 |
| IL | 9,634 | 1,458 | 1,691 |  | 15.1 | 17.6 | 17.7 |
| NC | 6,937 | 1,125 | 1,364 |  | 16.2 | 19.7 | 19.3 |
| TN | 4,462 | 797 | 1,004 |  | 17.9 | 22.5 | 23.3 |
| ND | 613 | 32 | 61 |  | 5.2 | 10.0 | 10.5 |
| ME | 1,152 | 66 | 128 |  | 5.7 | 11.1 | 11.2 |
| PA | 9,810 | 1,281 | 1,852 |  | 13.1 | 18.9 | 20.0 |
| NE | 1,619 | 156 | 290 |  | 9.6 | 17.9 | 16.3 |
| SD | 680 | 63 | 123 |  | 9.3 | 18.1 | 16.4 |
| KY | 3,477 | 812 | 1,164 |  | 23.4 | 33.5 | 34.8 |
| MI | 8,006 | 1,283 | 2,110 |  | 16.0 | 26.4 | 26.1 |
| SC | 3,319 | 621 | 964 |  | 18.7 | 29.0 | 29.5 |
| MO | 4,938 | 768 | 1,321 |  | 15.6 | 26.8 | 26.2 |
| OH | 9,300 | 1,711 | 2,761 |  | 18.4 | 29.7 | 29.8 |
| IA | 2,657 | 218 | 536 |  | 8.2 | 20.2 | 18.9 |
| IN | 5,202 | 876 | 1,518 |  | 16.8 | 29.2 | 29.7 |
| AL | 3,856 | 822 | 1,341 |  | 21.3 | 34.8 | 38.3 |
| OK | 3,227 | 599 | 1,078 |  | 18.6 | 33.4 | 35.6 |
| MS | 2,389 | 714 | 1,087 |  | 29.9 | 45.5 | 50.0 |
| AR | 2,256 | 523 | 904 |  | 23.2 | 40.1 | 42.5 |
| KS | 2,382 | 308 | 723 |  | 12.9 | 30.4 | 32.1 |
| WV | 1,607 | 303 | 619 |  | 18.9 | 38.5 | 41.3 |
| Source: 2020 Neighborhood Atlas ADI (v 3.2) retrieved from https://www.neighborhoodatlas.medicine.wisc.edu. Standardized ADI based on author's analysis of 2020 American Community Survey data. | | | | | | | |
| Notes: Counts for Atlas ADI v4.0 are not shown. Across states, the correlation between the Percent Eligible ADI 4.0 and Percent Eligible 3.2 is .998; the correlation between Percent Eligible Std and Atlas 3.2 is 0.799. | | | | | | | |

| Table A4b. Classification of Block Groups using 85th Percentile Cutoff Using Alternative Measures | | | | | | | |
| --- | --- | --- | --- | --- | --- | --- | --- |
| **b. Detailed Classification** | | | | | | | |
|  | **Counts of Block Groups above and Below 85th Percentile** | | | | | | |
|  | **Std ADI< 85** | |  | **Std ADI≥ 85** | |  |  |
|  | **Atlas<85** | **Atlas≥85** |  | **Atlas<85** | **Atlas≥85** | **Total** |  |
| Total | 181,008 | 17,332 |  | 17,331 | 20,447 | 236,118 |  |
| DC | 443 | - |  | 89 | - | 532 |  |
| CA | 21,202 | 119 |  | 3,648 | 262 | 25,231 |  |
| NV | 1,474 | 45 |  | 294 | 104 | 1,917 |  |
| RI | 675 | - |  | 85 | 7 | 767 |  |
| MA | 4,474 | 3 |  | 459 | 25 | 4,961 |  |
| CT | 2,352 | 15 |  | 258 | 35 | 2,660 |  |
| PR | 431 | 89 |  | 292 | 1,654 | 2,466 |  |
| NJ | 5,690 | 59 |  | 582 | 106 | 6,437 |  |
| NY | 12,231 | 639 |  | 1,819 | 611 | 15,300 |  |
| AZ | 3,569 | 205 |  | 508 | 374 | 4,656 |  |
| AK | 426 | 3 |  | 32 | 27 | 488 |  |
| OR | 2,656 | 34 |  | 205 | 29 | 2,924 |  |
| WA | 4,855 | 35 |  | 297 | 39 | 5,226 |  |
| HI | 960 | - |  | 49 | 2 | 1,011 |  |
| DE | 616 | 11 |  | 37 | 21 | 685 |  |
| MD | 3,563 | 56 |  | 197 | 166 | 3,982 |  |
| ID | 1,134 | 25 |  | 65 | 35 | 1,259 |  |
| UT | 1,873 | 15 |  | 74 | 17 | 1,979 |  |
| CO | 3,645 | 68 |  | 183 | 68 | 3,964 |  |
| NM | 1,022 | 116 |  | 154 | 278 | 1,570 |  |
| NH | 928 | 14 |  | 35 | 4 | 981 |  |
| MN | 4,144 | 153 |  | 212 | 60 | 4,569 |  |
| TX | 12,555 | 1,344 |  | 1,577 | 2,754 | 18,230 |  |
| VT | 515 | 6 |  | 12 | 2 | 535 |  |
| FL | 10,306 | 923 |  | 1,049 | 765 | 13,043 |  |
| LA | 2,645 | 396 |  | 435 | 704 | 4,180 |  |
| VA | 5,019 | 250 |  | 274 | 240 | 5,783 |  |
| WY | 417 | 16 |  | 14 | 3 | 450 |  |
| MT | 786 | 42 |  | 34 | 25 | 887 |  |
| GA | 5,337 | 603 |  | 515 | 855 | 7,310 |  |
| WI | 3,872 | 254 |  | 156 | 330 | 4,612 |  |
| IL | 7,203 | 973 |  | 740 | 718 | 9,634 |  |
| NC | 5,184 | 628 |  | 389 | 736 | 6,937 |  |
| TN | 3,221 | 444 |  | 237 | 560 | 4,462 |  |
| ND | 533 | 48 |  | 19 | 13 | 613 |  |
| ME | 987 | 99 |  | 37 | 29 | 1,152 |  |
| PA | 7,579 | 950 |  | 379 | 902 | 9,810 |  |
| NE | 1,274 | 189 |  | 55 | 101 | 1,619 |  |
| SD | 533 | 84 |  | 24 | 39 | 680 |  |
| KY | 2,143 | 522 |  | 170 | 642 | 3,477 |  |
| MI | 5,675 | 1,048 |  | 221 | 1,062 | 8,006 |  |
| SC | 2,217 | 481 |  | 138 | 483 | 3,319 |  |
| MO | 3,450 | 720 |  | 167 | 601 | 4,938 |  |
| OH | 6,248 | 1,341 |  | 291 | 1,420 | 9,300 |  |
| IA | 2,054 | 385 |  | 67 | 151 | 2,657 |  |
| IN | 3,523 | 803 |  | 161 | 715 | 5,202 |  |
| AL | 2,356 | 678 |  | 159 | 663 | 3,856 |  |
| OK | 2,049 | 579 |  | 100 | 499 | 3,227 |  |
| MS | 1,181 | 494 |  | 121 | 593 | 2,389 |  |
| AR | 1,248 | 485 |  | 104 | 419 | 2,256 |  |
| KS | 1,602 | 472 |  | 57 | 251 | 2,382 |  |
| WV | 933 | 371 |  | 55 | 248 | 1,607 |  |

| Table A5. Comparison of Factor Loadings and Scoring Coefficients, using 1990 and 2020 Data | | | |  |  |  |
| --- | --- | --- | --- | --- | --- | --- |
|  | **Factor Loadings** | |  | | **Scoring Coefficients** | |
| **Measure** | **1990** | **2020** |  | | **1990** | **2020** |
| Population below 150% of the poverty threshold, % | 0.916 | 0.849 |  | | 0.104 | 0.235 |
| Families below poverty level, % | 0.862 | 0.712 |  | | 0.098 | 0.107 |
| Income disparity | 0.826 | 0.699 |  | | 0.094 | 0.081 |
| Population aged ≥ 25 y with < 9 y of education, % | 0.750 | 0.529 |  | | 0.085 | 0.068 |
| Population aged ≥ 25 y with at least a high school diploma, % | -0.856 | -0.689 |  | | -0.097 | -0.180 |
| Employed persons aged ≥ 16 y in white-collar occupations, % | -0.772 | -0.664 |  | | -0.087 | -0.062 |
| Civilian labor force population aged ≥16 y unemployed, % | 0.712 | 0.358 |  | | 0.081 | 0.023 |
| Single-parent households with children aged < 18 y, % | 0.635 | 0.525 |  | | 0.072 | 0.051 |
| Owner-occupied housing units, % (home ownership rate) | -0.543 | -0.501 |  | | -0.062 | -0.076 |
| Households with more than 1 person per room, % | 0.613 | 0.315 |  | | 0.069 | 0.022 |
| Households without a motor vehicle, % | 0.775 | 0.385 |  | | 0.088 | 0.050 |
| Households without a telephone, % | 0.451 | 0.243 |  | | 0.051 | 0.019 |
| Occupied housing units without complete plumbing, % | 0.491 | 0.122 |  | | 0.056 | 0.013 |
| Median household income, $ | -0.863 | -0.817 |  | | -0.098 | -0.181 |
| Median home value, $ | -0.607 | -0.547 |  | | -0.069 | -0.091 |
| Median gross rent, $ | -0.690 | -0.575 |  | | -0.078 | -0.058 |
| Median monthly mortgage, $ | -0.680 | -0.607 |  | | -0.077 | -0.122 |
| Source: The 1990 factor loadings and scoring coefficients are from Singh (2003, Table 1). The 2020 estimates are from author's analysis of the 2020 American Community Survey. | | | | | | |
| Note: Singh's analysis is at the Census tract-level, the analysis of the 2020 is at the block group level. Income disparity is equal to the ln(median income<$10,000/median income>$50,000). | | | | | | |

| Table A6. Home Ownership, Home Values, Margins of Error and Missing Values | | | | | | | | | | |
| --- | --- | --- | --- | --- | --- | --- | --- | --- | --- | --- |
|  | | | | | | | | | | |
|  |  |  | **Median Home Values (ACS)** | | | |  | **ADI Ranking** | |  |
| **Decile** | **Ownership (%)** |  | **Estimate** | **MOE** | **Missing (%)** | **Imputed** |  | **v32** | **v40** | **Poor (%)** |
| 1 | 0.0-23.8 |  | 345,081 | 103,858 | 48.4 | 349,701 |  | 49.2 | 47.4 | 21.0 |
| 2 | 23.8-41.1 |  | 298,852 | 72,188 | 7.4 | 294,440 |  | 55.9 | 54.0 | 17.0 |
| 3 | 41.2-53.9 |  | 268,597 | 57,593 | 4.6 | 265,632 |  | 57.8 | 56.5 | 14.4 |
| 4 | 53.9-63.9 |  | 253,733 | 49,556 | 3.2 | 251,806 |  | 57.7 | 56.7 | 12.1 |
| 5 | 63.9-72.1 |  | 250,698 | 46,150 | 2.8 | 248,216 |  | 56.6 | 56.2 | 10.3 |
| 6 | 72.1-78.7 |  | 254,524 | 45,510 | 2.4 | 252,559 |  | 54.6 | 54.8 | 8.6 |
| 7 | 78.7-84.5 |  | 267,232 | 45,370 | 2.0 | 265,428 |  | 51.4 | 52.1 | 7.3 |
| 8 | 84.5-89.8 |  | 285,867 | 46,681 | 1.8 | 283,976 |  | 47.5 | 48.7 | 5.9 |
| 9 | 89.8-95.2 |  | 321,510 | 48,541 | 1.6 | 319,062 |  | 41.3 | 43.0 | 4.6 |
| 10 | 95.2-100.0 |  | 376,682 | 54,676 | 2.0 | 373,663 |  | 33.0 | 35.5 | 3.3 |
| Source: The 2020 Neighborhood Atlas ADI, version 3.2 and version 4.0 national rank percentiles were downloaded from https://www.neighborhoodatlas.medicine.wisc.edu. All other estimates were calculated by author using 2020 ACS block level data. | | | | | | | | | | |
| Note: With the exception of Version 4.0 of the Neighborhood Atlas ADI, all correlations are based on 236,136 block groups in the U.S., DC and Puerto Rico. MOE-Margin of Error. Imputed-Median home value after imputation from tract- and county-level. | | | | | | | | | | |

### Figure A2. Percentile Difference between Neighborhood Atlas ADI v32 and Simple ADI, using Alternative Median Income Measures


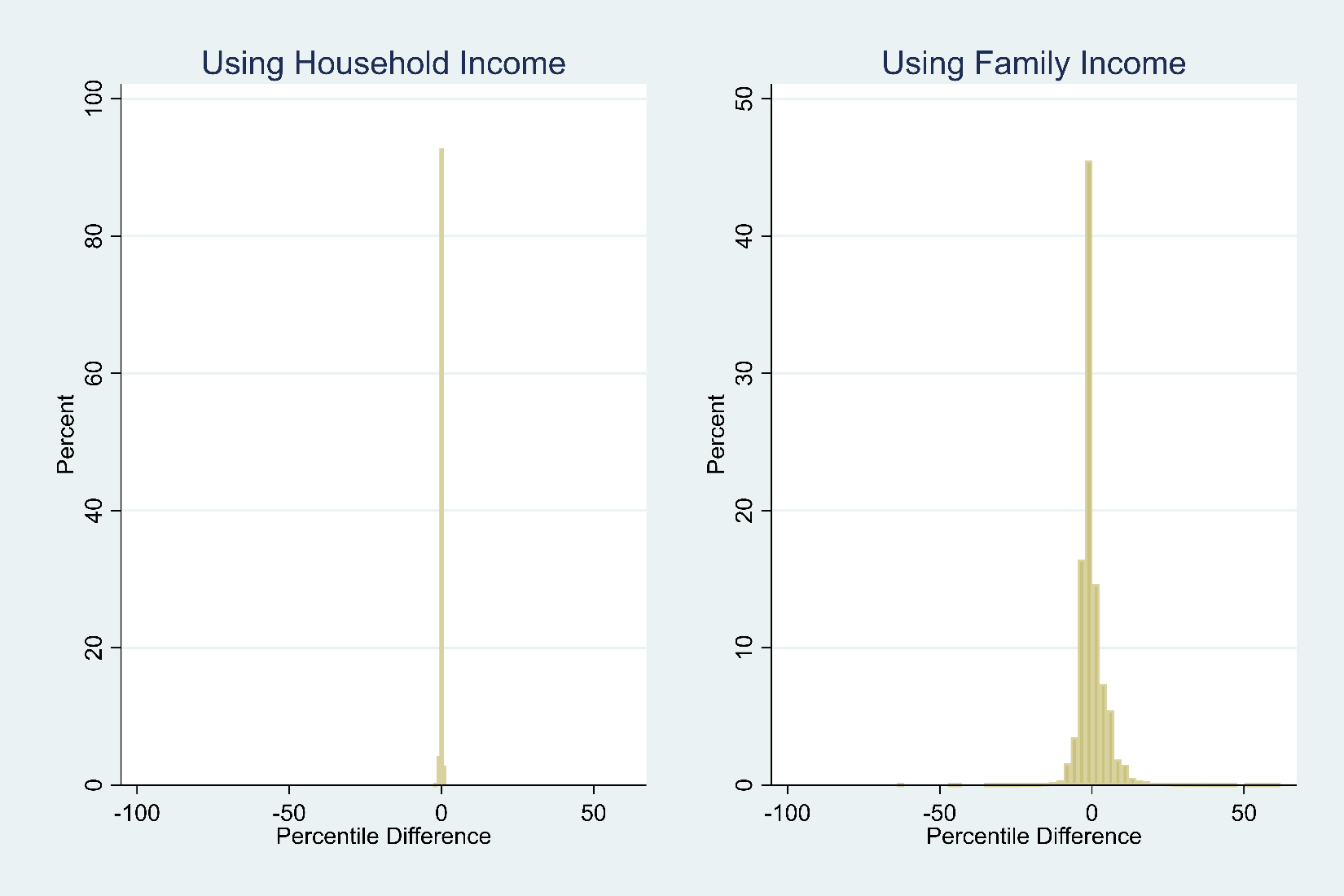


Source: 2020 Neighborhood Atlas ADI (v 3.2) retrieved from <https://www.neighborhoodatlas.medicine.wisc.edu>. Simple ADI based on author's analysis of 2020 American Community Survey data.

| Table A7. Select Characteristics of Least Deprived Decile using Neighborhood Atlas ADI, by Standardized ADI Deciles | | | | | | | |
| --- | --- | --- | --- | --- | --- | --- | --- |
| **Deciles Standardized ADI** | **Frequency** | **Median Home** | **Median Income** | **Poverty** | **Owner Occupied** | **Standardized ADI** | **NA ADI** |
| 1 | 14,205 | $953,572 | $166,221 | 1.8% | 80.7% | 4.3 | 4.88 |
| 2 | 3,632 | $829,209 | $112,662 | 3.8% | 59.7% | 14.7 | 6.44 |
| 3 | 1,722 | $835,636 | $96,725 | 5.3% | 49.1% | 25.1 | 6.53 |
| 4 | 1,026 | $844,575 | $90,032 | 7.4% | 43.5% | 35.1 | 6.49 |
| 5 | 667 | $855,776 | $81,500 | 9.2% | 37.6% | 45.4 | 6.5 |
| 6 | 570 | $861,297 | $74,611 | 10.9% | 32.6% | 55.6 | 6.47 |
| 7 | 517 | $884,231 | $67,893 | 13.7% | 29.5% | 65.7 | 6.34 |
| 8 | 470 | $898,120 | $59,459 | 18.3% | 23.7% | 75.5 | 6.33 |
| 9 | 427 | $908,812 | $50,986 | 23.2% | 17.3% | 85.7 | 6.17 |
| 10 | 403 | $934,997 | $33,427 | 35.5% | 8.9% | 95.4 | 6.35 |
| Total | 23,639 | $912,414 | $136,404 | 4.6% | 66.5% | 16.9 | 5.5 |
|  | | | | | | | |
